# Role of Endothelial and Mesenchymal Cell Transitions in Heart Failure and Recovery Thereafter

**DOI:** 10.3389/fgene.2020.609262

**Published:** 2021-01-15

**Authors:** Guangyu Wang, Ana Sofia Cruz, Keith Youker, Hernan G. Marcos-Abdala, Rajarajan A. Thandavarayan, John P. Cooke, Guillermo Torre-Amione, Kaifu Chen, Arvind Bhimaraj

**Affiliations:** ^1^Center for Bioinformatics and Computational Biology, Houston Methodist Research Institute, Houston, TX, United States; ^2^Center for Cardiovascular Regeneration, Houston Methodist Research Institute, Houston, TX, United States; ^3^Department of Cardiovascular Sciences, Weill Cornell Medicine, Cornell University, Houston, TX, United States; ^4^Houston Methodist DeBakey Heart and Vascular Institute, Houston Methodist Hospital, Houston, TX, United States; ^5^Tecnologico de Monterrey, Escuela de Medicina y Ciencias de la Salud, Medicina Cardiovascular y Metabolómica, Monterrey, Mexico

**Keywords:** heart failure, cardiac recovery, endothelial to mesenchymal transition, mesenchymal to endothelial transition, gene expression

## Abstract

**Background:** Mechanisms of myocardial recovery are not well elucidated.

**Methods:** 3-month-old C57/BL6 mice were treated with Angiotensin-II infusion and N (w)-nitro-L-arginine methyl ester in drinking water to induce HF at 5 weeks. These agents were discontinued, and animals studied with echocardiographic, histological and genetic assessment every 2 weeks until week 19. mRNA was extracted from these samples and human pre-post LVAD samples.

**Results:** Histologic and echo characteristics showed progressive worsening of cardiac function by week 5 and normalization by week 19 accompanied by normalization of the transcriptional profile. Expression of 1,350 genes were upregulated and 3,050 genes down regulated in HF compared to controls; during recovery, this altered gene expression was largely reversed. We focused on genes whose expression was altered during HF but reverted to control levels by Week 19. A gene ontology (GO) analysis of this cohort of genes implicated pathways involved in EndoMT and MEndoT. The cohort of genes that were differentially regulated in heart failure recovery in the murine model, were similarly regulated in human myocardial samples obtained pre- and post-placement of a left ventricular assist device (LVAD). Human end stage HF myocardial samples showed cells with dual expressed VE-Cadherin and FSP-1 consistent with cell fate transition. Furthermore, we observed a reduction in fibrosis, and an increase in endothelial cell density, in myocardial samples pre- and post-LVAD.

**Conclusions:** Cell fate transitions between endothelial and mesenchymal types contribute to the pathophysiology of heart failure followed by recovery.

## Introduction

The pathophysiology of heart failure includes an inciting insult (genetic mutations, hemodynamic overload, myocyte destruction, etc.) that starts a cascade of compensatory adaptive and maladaptive mechanisms. Adaptive mechanisms often are unable to completely undo the response to the injury and restore a state of normalcy hence leading to the histological phenotype of cardiac hypertrophy and fibrosis. A strategy to discover mechanisms and pathways of repair and recovery could lead to novel therapeutic avenues to facilitate adaptation and recovery. After the onset of systolic dysfunction, cardiac recovery with neurohormonal blockade can occur in a subset of patients. Furthermore, chronic unloading of the heart with durable ventricular assist devices can improve cardiac function and structure. However, the mechanisms that are directly responsible for such recovery have not been clearly elucidated (Margulies et al., 2005). Lack of a controlled experimental setting combined with significant biological variations of clinical subjects has made it difficult to gain insight into specific mechanisms. We present here a non-ischemic murine model of heart failure recovery that is associated with histological and transcriptional evidence of cardiac recovery. We correlate the molecular signal from these experimental tissues with those from human myocardial samples obtained pre- and post-placement of a left ventricular assist device (LVAD).

## Methods: Murine Experiment

### Mouse Model

**Figure 1:**
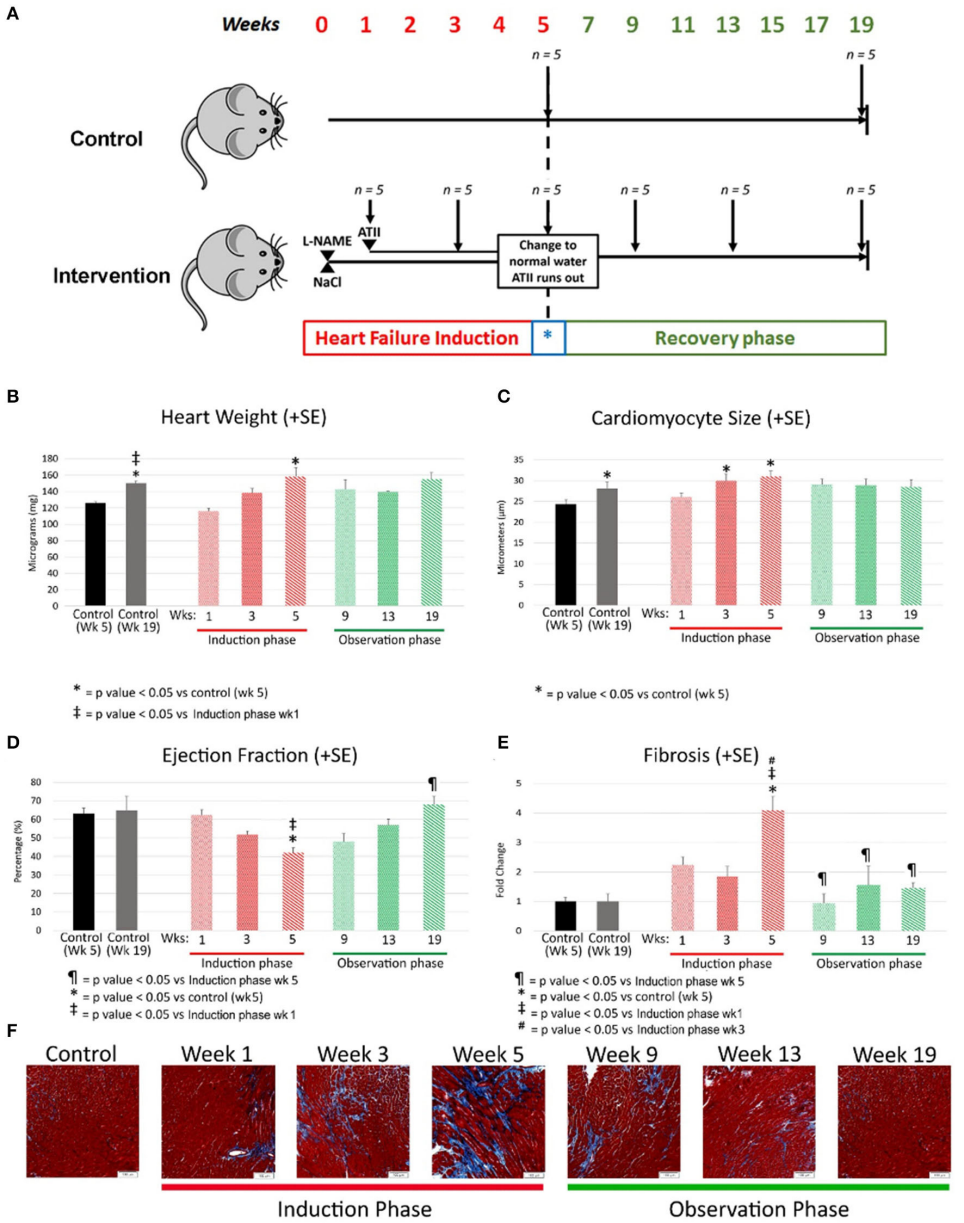
**(A)** Experimental model where C57/BL6 mice underwent heart failure induction over first 5 weeks followed by removal of heart failure-inducing agents (marked with a blue *) with a passive observation phase up to a total of 19 weeks. Each group had 5 mice and were sacrificed at the time points indicated by a downward arrow. L-NAME and NaCl were administered in drinking water and angiotensin II delivered by osmotic mini-pumps. L-NAME, NG-nitro-L-Arginine Methyl Ester; NaCl, Sodium Chloride; ATII, Angiotensin II. **(B–E)** Morphological changes at various time points 2 weeks apart during heart failure induction and recovery phase after removal of heart failure-inducing agents. The bar graphs represent heart weight in milligrams **(B)**, cardiomyocyte size in micrometers **(C)**, ejection fraction as percentage **(D)** and fibrosis as fold change **(E)**. Red represents the heart failure induction phase and green represents the observation phase after removal of heart failure-inducing agents. Controls are represented in different colors to represent age matched controls for both time points. **(F)** Are representative photomicrographs of mouse cardiac with Masson's trichrome staining reflecting the gradual increase of fibrosis (by week 5) followed by a resolution of the same during the observation phase (recovery). L-NAME, NG-nitro-L-Arginine Methyl Ester; NaCl, Sodium Chloride; HF, Heart failure; Wks, weeks.

A modified model from Oestreicher et al. was used for the heart failure induction (Oestreicher et al., 2003). Physiological details including biomarkers, echo and hemodynamics are detailed in previous work from our group (Cordero-Reyes et al., 2016; Hamilton et al., 2016). In brief, 3-month-old male wild-type (WT; strain C57BL/6J; Harlan Laboratories, Indianapolis, IN) mice were randomly assigned to two groups: control and intervention (Figure 1A) and studied at regular intervals from week 1 to 19 (*n* = 5 at each time point in each experimental group). Mice in the intervention group were given drinking water containing 0.3 mg/mL of L-NAME (Sigma-Aldrich, St. Louis, MO) and 1% NaCl (Sigma-Aldrich) ad libitum. After 1 week, mice were anesthetized with inhaled isoflurane and subcutaneous osmotic minipumps (ALZET model 1004; DURECT Corporation ALZET Osmotic Pumps, Cupertino, CA) were implanted to deliver angiotensin-II (Ang-II; Sigma-Aldrich) at a rate of 0.7 mg/kg per day for 4 weeks. At the end of this 5-week period (Heart failure induction phase), mice develop a phenotype of heart failure including cardiac hypertrophy, fibrosis and a reduction of ejection fraction. At the 5-week time point, the Alzet pumps were depleted of angiotensin-II, and the L-NAME treatment was terminated. Mice were observed without drug treatment for up to week 19 (Recovery Phase). All mice were housed in micro isolation cages under 12-h: 12-h light/dark cycle and were fed a standard chow diet (Harlan Teklad, 2920).

Animals were euthanized under deep inhalatory anesthesia, followed by cervical dislocation and exsanguination. Hearts were immediately excised, weighed and dissected for left ventricle separation and sectioning. To obtain enough sample material for each assay and ensure reproducibility, the experiment was repeated in four separate cohorts. All experiments were performed with approval of the Houston Methodist Research Institute Institutional Animal Care and Use Committee (Houston, TX) and conformed to the Guide for the Care and Use of Laboratory Animals published by the US National Institutes of Health (NIH Publication No. 85-23, revised 1985).

### Sample Collection

Mouse hearts were rapidly removed and sectioned at the level of mid heart. Apical portions were flash-frozen with liquid nitrogen and kept at −80°C for polymerase chain reaction (PCR) studies and RNA sequencing. The basal portions were fixed in 2% paraformaldehyde, processed by dehydrating in graded alcohol to xylene followed by paraffin embedding and cut into 5-micron sections.

### Histological Analysis

To quantify fibrosis, sections were stained using a Masson's trichrome kit (Sigma-Aldrich), according to manufacturer's instructions. Slides were then cover slipped and photomicrographs taken with the 10X objective using an Olympus AX70 microscope (Olympus, Tokyo, Japan). Cross-sectional pictures at the mid-heart level were taken and analyzed by a blinded observer for fibrosis using Cell Sense Dimension Software from Olympus (Olympus, Tokyo, Japan). A user-independent automatically computerized color cube-based selection criterion was used to denote positive staining (within the color spectrum of blue dye) and stained/unstained areas were measured. The results expressed are the average percent tissue area (pixels) stained by the dye. To measure cardiomyocyte size, myocardial sections were stained with H&E using standard protocols. Slides were then cover slipped with photomicrographs taken at 20x. Measurement was done as previously reported (Bruckner et al., 2001) by measuring myocyte diameter at the level of the nucleus by a blinded observer.

### Echocardiography

All mice underwent transthoracic echocardiography prior to sacrifice under light anesthesia using inhaled isoflurane. Transthoracic echocardiography was performed and analyzed as previously reported (Respress and Wehrens, 2010) using Vevo770 high-resolution imaging system equipped with a 30-MHz transducer (Visualsonics, Toronto, Ontario, Canada). The heart was visualized in B-mode from parasternal short-axis and apical view. Two-dimensional targeted M-mode echocardiographic images were obtained at the levels of the papillary muscles from the parasternal short-axis view for measurements of LV wall thickness, LV end-diastolic diameter (LVEDD) and LV-end systolic diameter (LVESD) using at least three consecutive heart beats. All M-mode images were read from the same position. The investigator performing the echocardiograms was different from the blinded investigator who analyzed the images. Ejection Fraction (EF) was calculated as [(LVEDV-LVESV)/LVEDV] X 100.

### RNA Sequencing

RNA sequencing (Novo gene, Illumina Genome Network partner) was performed after RNA isolation from frozen tissue, poly (A) tail enrichment and library production (Novo gene, Illumina Genome Network partner). The RNA-seq results were analyzed at the Center for Bioinformatics and Computational Biology (CB^2^) at Houston Methodist Research Institute (HMRI).

### Gene Differential Expression Analysis and Gene Ontology (GO) Analysis

The Top Hat program (v2.0.12) with default parameters was used to align paired-end RNA-seq reads to the mouse genome (Mus musculus MGSCv37). Cuffdiff (v2.0.12) was used to calculate gene expression level and significance of differential expression. We used a *p* value < 0.05 as the threshold to select differentially expressed genes. We used DAVID (https://david-d.ncifcrf.gov) to calculate *p* value for each GO term with a modified Fisher's exact test, and used a *p* value < 0.05 as the threshold to select significantly enriched GO terms.

## Human Myocardial Samples

### Heart Failure Samples

Left ventricular tissue was collected from 25 consecutive end-stage heart failure patients (apical core at the time of Left Ventricular Assist Device placement or section of LV at the time of heart transplant). Fresh tissue was obtained directly from the surgeon, and samples were immediately dissected, and tissues were divided for either freezing or paraffin-embedding. Frozen tissue was flash-frozen in liquid nitrogen and immediately stored at −80°C for RNA and protein analysis. Paraffin-embedded tissue was immediately fixed in 2% paraformaldehyde; tissue samples were then dehydrated in a step-wise fashion using graded alcohols, after which they were cleared in xylene and paraffin-embedded by use of standard protocols. Five-micrometer sections were cut, collected on slides and rehydrated for histology and immunohistochemistry. This is based on an institutional protocol approved by the Houston Methodist Hospital Institutional Review Board [IRB (2)0511-0100].

### Dual Staining Cells

We stained samples with Anti-VE cadherin (Santa Cruz Biotechnology) as a marker of endothelial cells and Anti-FSP1 as a marker of fibroblasts. Presence of both markers (dual staining cells) may be reflective of cellular transition during the EndoMT process. Dual staining cells were counted per HPF.

### Paired Samples

Paired samples of myocardial tissue were collected from 14 patients during LVAD insertion (pre-LVAD) and at the time of LVAD removal during heart transplantation. For the post-LVAD sample, LV samples were obtained at least 2 cm away from LVAD insertion site to avoid surgically-related scar tissue. Tissues were frozen and stored at −80°C for RNA isolation and adjacent samples embedded in paraffin using standard techniques. Pre and post LVAD (obtained closest to surgical intervention) echocardiograms were interpreted to document cardiac function by a blinded echocardiographer, and parameters and calculations were reported following standard clinical American Society of Echocardiography guidelines.

### Immunohistochemistry

Myocardial fibroblasts or endothelial cell counts were quantified by staining for FSP-1 or VE-Cadherin (Cell Signaling Technology, Danvers, MA), respectively. Four low power fields were analyzed, and the results expressed as mean ± SD. The analysis was done by observers who were blinded to the sample source.

### Fibrosis

To quantify fibrosis, sections were stained using a Masson's trichrome kit (Sigma-Aldrich), according to manufacturer's instructions after rehydrating process through xylene and alcohol. Slides were then cover slipped and analyzed at x10 magnification using an Olympus AX70 microscope (Olympus, Tokyo, Japan). Pictures were taken of all regions of the heart and analyzed for fibrosis using Cell sense Dimension Software from Olympus (Olympus, Tokyo, Japan). A user-independent computerized color cube-based selection criterion were used to denote positive staining (within the color spectrum of blue dye) and stained/unstained areas were measured. The results expressed are the average percent tissue (pixels) stained by the dye. Analysis was performed by a single investigator blinded to the samples.

### Gene Expression

Total mRNA was isolated from human cardiac tissue using RNA-binding columns per manufacturer's protocol. RNA was reverse transcribed into cDNA using a kit per manufacturer's directions. Sense and antisense primers were chosen from the RTPrimer DB public database for human. Reactions were aliquoted into 96-well plates, plates were sealed, and samples were amplified for 40 cycles of 10 s at 95°C, 30 s at 55°C and 10 s at 72°C. Real-time PCR was performed in a MyIQ5 iCycler (Bio-Rad). All samples were measured in triplicates and normalized to GAPDH controls run on the same plate for each human cDNA sample that was tested. Gene expression levels were calculated by using [2^Ct(gene)^/2^Ct(GAPDH)^] × 1,000 to standardize to GAPDH and expressed as relative transcript numbers.

### Western Blot Analysis

Protein lysates were prepared from cardiac tissue as described previously (Thandavarayan et al., 2009). The total protein concentration in the samples was measured by the bicinchoninic acid method. Proteins were resolved by electrophoresis and transferred to a nitrocellulose filter. Then the blots were blocked with 5% non-fat dried milk and washed with tris buffered saline. After incubation with the primary antibodies and horseradish peroxidase coupled secondary antibodies (Santa Cruz Biotechnology) the bands were detected by using chemiluminescence developing agents (Super Signal West Pico, Thermo Scientific). The images of the protein bands were taken with a BIO-RAD ChemiDoc imaging system.

### Comparative Analysis of Gene Changes From Mouse Model With Human Gene Expression Data

The gene expression changes in the mouse samples from heart failure to recovery were compared at a global level to changes in the human pre and post LVAD myocardial gene expression using gene set enrichment analysis (GSEA) (Subramanian et al., 2005).

### Statistical Analysis

Data are presented as mean ± standard error mean and one-way analysis of variance (ANOVA) was performed to identify differences between multiple groups. A two-tailed *p* = 0.05 was used as the significance cutoff for all tests. Analyses were done using Graph Pad Prism software (Graph Pad Software Inc., La Jolla, CA) or STATA13 (StataCorp LP, College Station, TX).

## Results

### Morphological Characterization of Recovery From Heart Failure

#### Heart Weight

Heart weight was increased by week 5 in comparison to age matched controls (158 ± 27 mg; *p* value < 0.05) and when compared to week 1. The heart weight regressed to control levels during the recovery phase (Age matched control: 150 ±4.7 mg; week 19 observation: 155 ± 14 mg; *p* value = 0.51). The heart weight did increase with age in the control group reflective of changes with aging (Figure 1B).

#### Cardiomyocyte Size

Cardiomyocyte size is a marker of myocardial hypertrophy, which increased early in the development of heart failure (control: 24.4 ± 2.1 micrometers; week 3: 30.2 ± 2.4 micrometers; *p* value = 0.055) and remained high at week 5 as heart failure progressed (30.4 ± 3.1 micrometers; *p* value < 0.05 vs. control). Myocyte size regression was evident during the recovery phase with the average cardiomyocyte size at Week 19 being no different than that of age-matched controls (control: 28.1 ± 3.0 micrometers; week 19: 28.5 ± 3.5 micrometers; *p* value = 0.8) (Figure 1C).

#### Echocardiography

EF decreased early in the progression of heart failure from 63 ± 8% in control to 52 ± 4% by week 3 (*p* value < 0.05) with a further drop by week 5 (42 ± 7; *p* value < 0.001 vs. control; *p* value = 0.09 vs. week 3). EF normalized during the recovery phase (week 19: 68 ± 8%; control: 65 ± 11%; *p* value = 1) (Figure 1D). Fractional shortening measurements tracked similarly to ejection fraction (Data not shown).

#### Fibrosis

Histological quantification of interstitial myocardial collagen deposition (fibrosis) increased through week 3 (control: 2.4 ± 0.7%; week 3: 4.4 ± 1.6%; *p* value = 0.08 vs. control) and peaked at week 5 (9.1 ± 0.04%). Fibrosis was greater at week 5 compared to week 3 (*p* value < 0.01) and 5-week age matched controls (*p* value < 0.01). The quantity of fibrosis decreased early during the recovery phase (Figure 1E). Representative pictures of the fibrosis staining are shown in the bottom row of Figure 1F.

### Transcriptional Profile Assessment

#### Global Gene Expression

**Figure 2:**
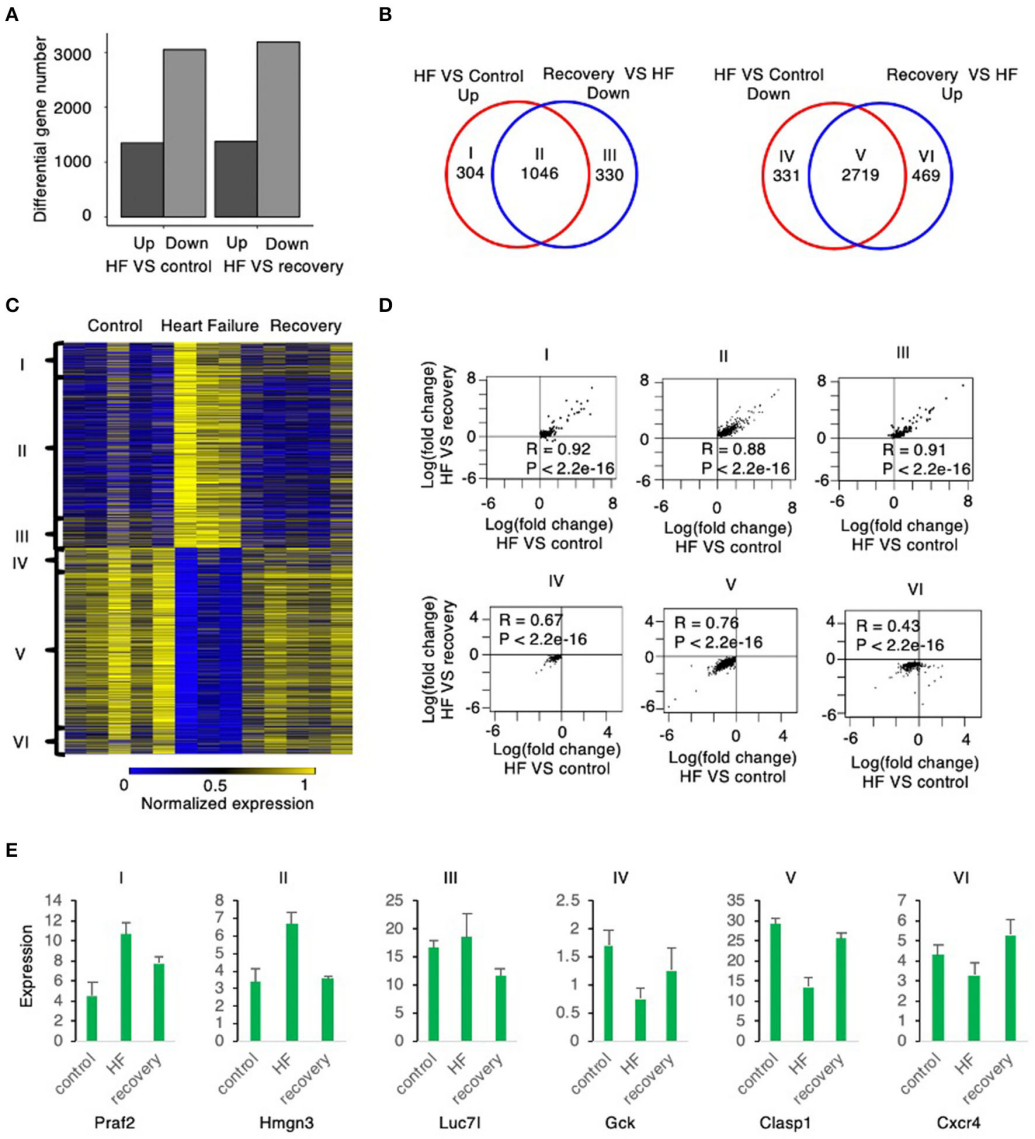
Global gene expression changes from mouse myocardium samples from control to heart failure and recovery. **(A)** The number of up and down regulated genes in heart failure relative to control are similar to the number of up and down regulated genes in heart failure relative to recovery suggesting that recovery is similar to control state. **(B)** The up regulated genes in heart failure relative to control correlate highly with the down regulated genes in recovery relative to heart failure (group II). The down regulated genes in heart failure relative to control correlate highly with the up regulated genes in recovery relative to heart failure (group V). Group I and group IV reflects number of genes that are up and down regulated, respectively, in HF compared to control (without a correlation to those reverting back to control state in recovery); Similarly, Group III and group VI are No. of genes that are down and up regulated, respectively, in recovery compared to HF (without a correlation between control and HF). **(C)** The heatmap shows the expression level of the six groups defined above. **(D)** The scatter plot of each group shows the expression change in heart failure relative to control is strongly correlated with the expression change in heart failure relative to recovery. **(E)** The boxplots show the expression level of example genes in each of the 6 groups described above.

With the induction of heart failure (at week 5), there were 1,350 genes with up regulated expression and 3,050 genes with down regulated expression (Figure 2A). With recovery from HF (week 19), a similar number of genes were differentially regulated: 1,376 with down regulated expression and 3,188 genes up regulated during recovery compared to week 5 (HF) (Figure 2A).

The Venn diagrams in Figure 2B define six groups of genes based upon the pattern of their expression during the induction of, and the recovery from, heart failure. Group I (304 genes) were significantly upregulated during heart failure induction but show no significant down regulation during recovery, whereas Group II (1,046 genes) were significantly up regulated during heart failure induction and reverted to significant down regulation during recovery. Group III (330 genes) did not show significant up regulation during heart failure induction but was significantly down regulated during recovery. Group IV (331 genes) was significantly downregulated during induction of heart failure but not significantly up regulated during recovery. Group V (2,719 genes) was downregulated during heart failure induction and reverted to significant up regulation during recovery; whereas Group VI (469 genes) did not show significant down regulation during heart failure but was significantly upregulated during recovery.

Most genes that were differentially regulated in heart failure were reverted to basal levels during recovery. Of the genes with upregulated expression during the induction of heart failure 1,046, 77.5% (Group II) were normalized to control levels after recovery phase (week 19). Of the genes with downregulated expression during the induction of heart failure, 2,719 (89.1%; Group V) were normalized to control levels during the recovery phase. The changes of the genes from control to heart failure when compared to changes from heart failure to recovery were highly correlated (Figure 2D). Examples of genes corresponding to each of these groups is shown in Figure 2E.

#### Assessment of Functional Genetic Changes

**Figure 3:**
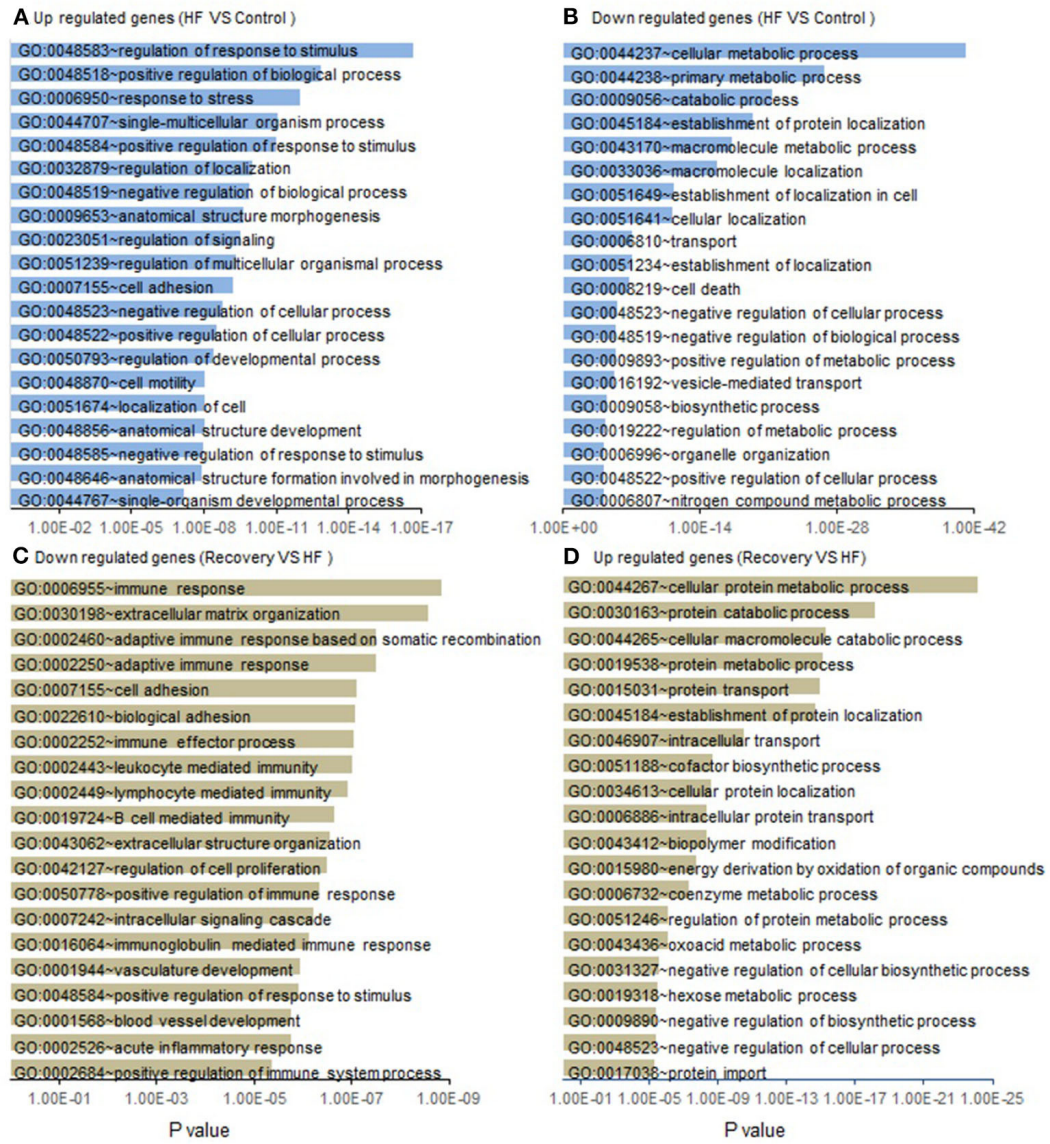
List of functional GO pathways of gene changes from control to heart failure and recovery. Assessment of gene expression changes in Heart failure (Week 5) compared to controls show **(A)** 1,350 Up regulated genes enrich into a total of 95 GO pathways of which the top 20 most significant are shown; **(B)** 3,050 down regulated genes enrich into a total of 46 GO pathways and top 20 most significant are shown. Functional GO pathway assessment of gene expression changes in Recovery (Week 19) compared to Heart failure (Week 5). **(C)** 3,188 down regulated genes enrich into a total of 185 GO pathways of which the top 20 most significant are shown. **(D)** 1,376 up regulated genes enrich into a total of 146 GO pathways and top 20 most significant are shown. *p*-value is statistical significance.

Gene Ontology (GO) enrichment analysis of those genes with up regulated expression in heart failure (week 5) compared to control enriched into 95 GO pathways. Those genes with down regulated expression enriched into 46 GO pathways. Similarly, gene expression at week 19 when compared to week 5 showed that the genes with up regulated expression enriched into 185 GO pathways and those with down-regulated expression enriched into 384 GO pathways. The top 20 (numerically most significant) of these GO pathways are presented in Figure 3 as a potential representation of these pathways and the entire list is presented in the Supplementary Tables 1–4.

**Figure 4:**
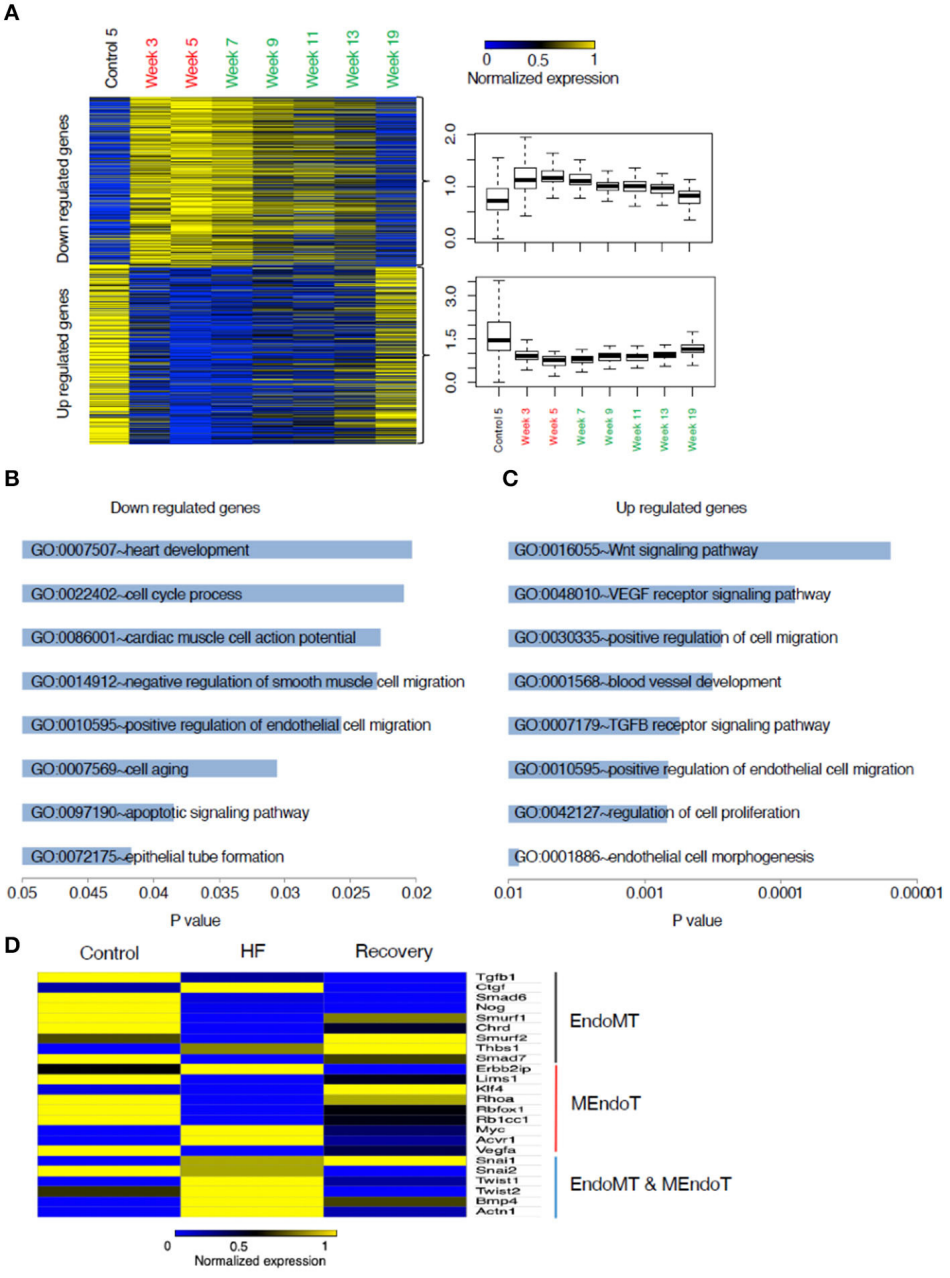
Analysis of genes that changed from control to heart failure and reverted to control state in recovery. **(A)** Heat map of these genes categorized into those up regulated in heart failure compared to control followed by reverting to a lower expression in recovery and those down regulated in heart failure compared to control followed by reverting back up in the recovered state. **(B)** List of the most relevant biologically meaningful GO pathways that suggested epithelial/endothelial and mesenchymal transitions into which the genes that were up regulated in heart failure cohort into and those **(C)** representing genes that were down regulated during heart failure. **(D)** Relative changes in gene expression of a list of 24 genes that were identified from literature search as those that play a role in endothelial and mesenchymal cell transitions. The heat map represents changes in gene expression from control to week 5 (representing heart failure) and week 19 (representing recovery). Yellow represents a relative increased expression and blue a decreased expression.

To understand the mechanisms underlying the reversion to normalcy from the heart failure state, we focused on those genes that were differentially regulated in HF but reverted to basal levels during recovery (Groups II and V) (Figure 4A). Functional GO pathway analysis showed that the genes in Group II cohorted into 298 GO pathways; whereas the genes in Group V cohorted into 333 GO pathways. Of these pathways, 30 pathways in the upregulated genes and 20 in the down regulated genes seemed to be related to processes that contribute to epithelial or endothelial and mesenchymal transitioning (Figures 4B,C). The remaining GO pathways represented metabolic changes, DNA repair, RNA metabolism, mitochondrial and myocyte interactions.

As the processes in endothelial and mesenchymal transitions are relatively unexplored in the context of heart failure recovery, we decided to focus on genes regulating these processes. While EMT and EndoMT are two distinct phenomena, many of the processes related to the cell fate transitions at the cellular level have been suggested to be similar (Kovacic et al., 2019) and hence we included certain genes that have been best studied in EMT but might not be well validated specifically in EndoMT. Our literature search generated a set of 24 genes commonly identified as having a role in EMT/EndoMT and MEndoT. The relative expression of these genes comparing control, week 5 and week 19 is shown in the heat map presented in Figure 4D. The expression of most of the pro-fibrotic and EndoMT mediating genes like CTGF, SNAI1, SNAI2, Twist 1 and 2, BMP4, and Actn1 are upregulated during heart failure and most of these are down-regulated at Week 19 (recovery). SNAI1 was persistently upregulated even in recovery supporting a recent suggestion (Barrallo-Gimeno and Nieto, 2005) that SNAI1 (also called SNAIL) plays a role in cell motility and migration and hence possibly have a broader role in cell fate transitions without a specific directionality. This process could hence contribute toward not just the progression from normal to a heart failure state but also from failure to recovery. Smurf2, Thbs1, Klf4 seem to be uniquely up regulated at Week 19 suggesting their contribution toward recovery.

### Correlation With Human Myocardial Samples

**Figure 5:**
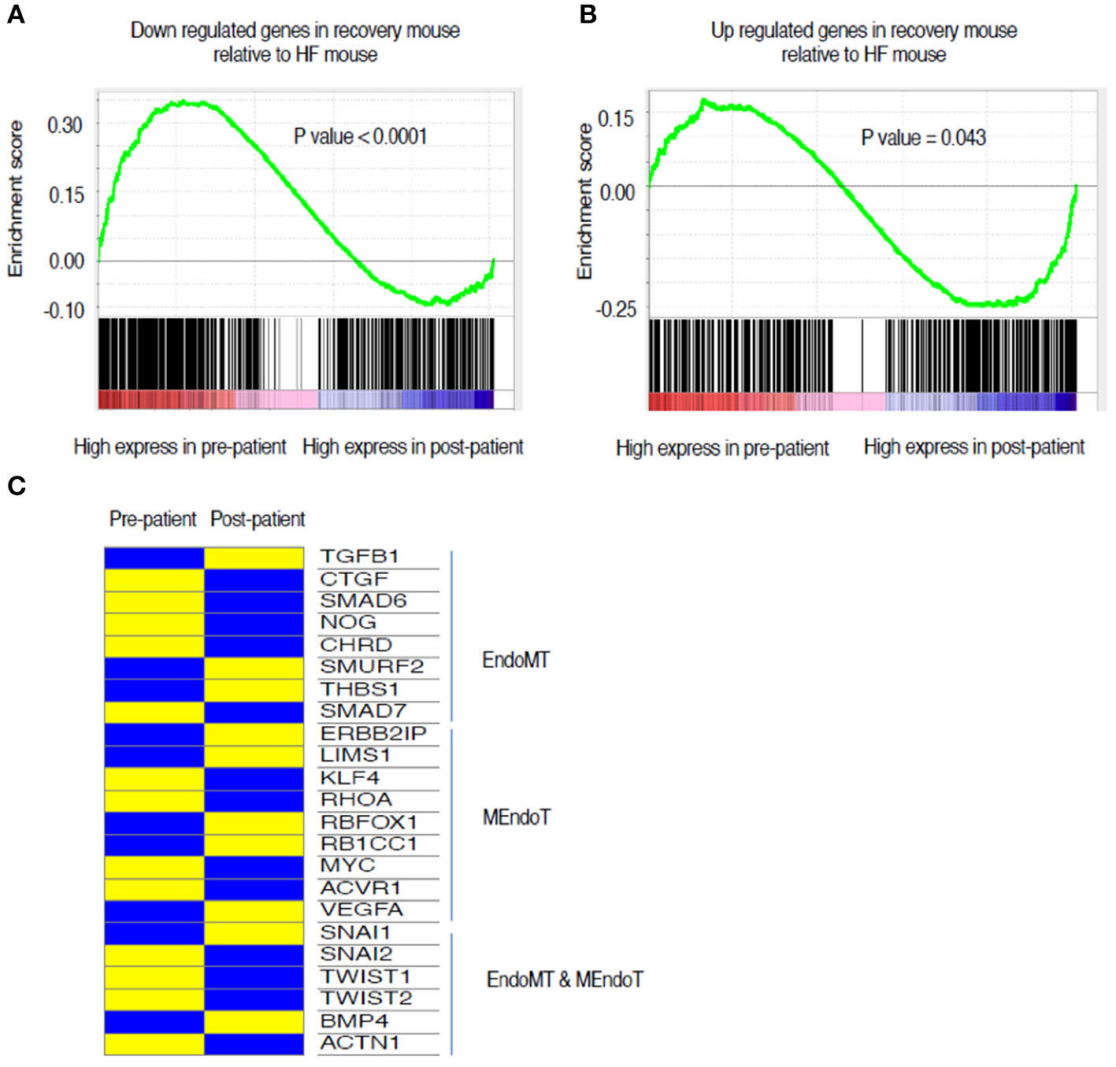
Comparison of directionality of gene expression changes between HF and recovery stages of mouse myocardium in relation to changes in gene expression from pre- and post LVAD human myocardial samples. The green line is representing mouse gene expression while the lower column of black lines are representing gene expression data from pre and post LVAD paired human myocardial samples. **(A)** The genes that are up regulated in HF mouse compared to recovery are also skewed toward higher expression in the pre-LVAD human myocardial samples (representing chronic heart failure). **(B)** The genes that are Up regulated in recovery in the mouse compared to HF, are expressed more in the post LVAD human samples in comparison to the pre-LVAD samples. **(C)** Relative changes in gene expression of the list of 24 genes that were identified from literature search and assessed in the mouse samples (Figure 4D). The heat map represents changes in gene expression compared between the pre-LVAD myocardium (representing heart failure) to post LVAD myocardium of the same individual (representing recovery). Yellow represents a relative increased expression and blue a decreased expression HF, Heart failure; LVAD, Left Ventricular Assist Device.

**Figure 6:**
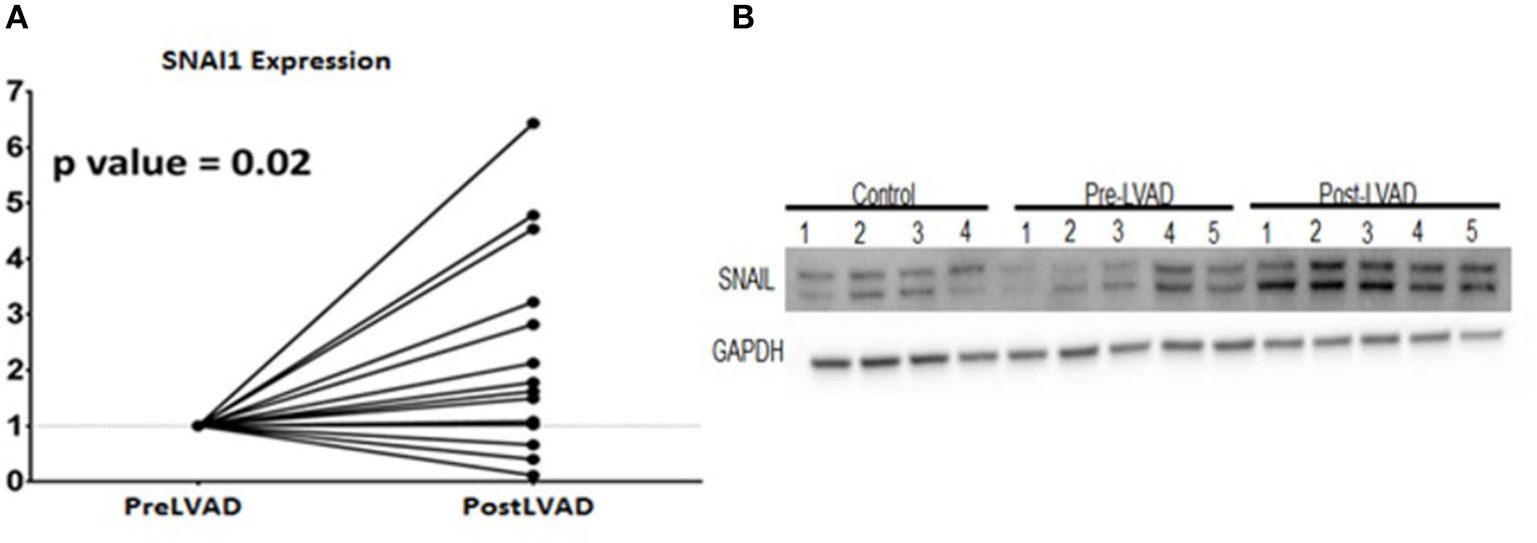
Expression changes of SNAI 1 in pre- LVAD and post LVAD human myocardial samples showing **(A)** RT-PCR of SNAI1 shows the fold increase of gene expression in the post LVAD samples compared to pre-LVAD samples; **(B)** Western Blot showing similar protein expression that increases from pre-LVAD to post-LVAD samples.

To assess the translational validity of genetic changes found in the mouse hearts as described above, the directionality of the gene expression changes from heart failure (week 5) to recovery (Week 19) were compared to the gene expression changes from human myocardial samples of pre-LVAD myocardium (representative of heart failure) and post-LVAD myocardium (representative of recovery) obtained as paired samples from 14 patients who were receiving a LVAD and subsequently underwent a heart transplantation. Mean age was 53 years, 77% were male, 54% African American, 46% had ischemic cardiomyopathy, 30% diabetes and 69% hypertension. The average time of being supported with the LVAD was 295 days (100–1,426 d). The pre- and post LVAD echocardiographic characteristics of these patients suggested that the left ventricle had partially recovered function and structure, as the ejection fraction improved from 17 to 24.3% (*p* = 0.03) and the left atrial volume index decreased from 50.6 to 24.1 ml/m^2^ (*p* < 0.001). The genes that were down regulated in the recovery phase of the mouse model compared to the heart failure state were highly expressed in the pre-LVAD human samples in relation to the post-LVAD samples (Figure 5A). Those genes that were up regulated in the recovery phase of the mouse model were highly expressed in post-LVAD human samples (Figure 5B). We then assessed the EMT/EndoMT and MEndoT specific genes that were analyzed in the mouse samples for relative change in expression in the human myocardial gene expression data (Figure 5C) showing that many of the gene changes that were observed in the mouse samples (Figure 4D) also occurred in a similar direction in human samples. Specifically of note was gene expression of SNAI1 in pre and post LVAD myocardial samples which seems to parallel the changes found in the mouse model with an increase of myocardial SNAI1 during recovery (Figure 6A). Consistent with the SNAI1 gene expression, we also detected an increase in protein expression of SNAI1 by western blot in the remodeled human heart (Figure 6B).

**Figure 7:**
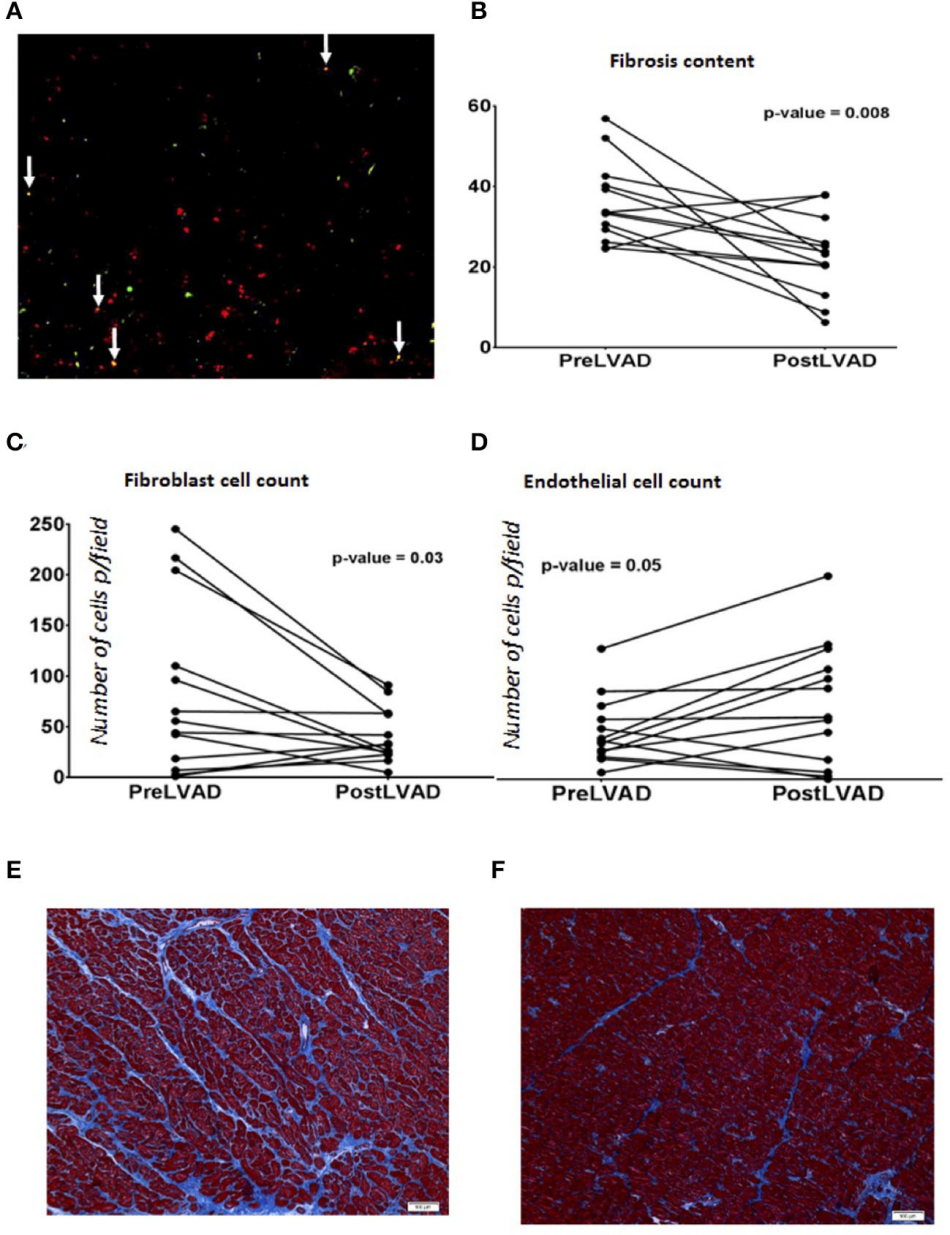
**(A)** Immunofluorescence staining of endothelial and mesenchymal cells in human end stage heart failure myocardial sample under Low power magnification (100X) showing many yellow cells (white arrows) reflecting cells in transition expressing both endothelial marker VE-Cadherin (green) and mesenchymal marker, FSP-1 (Red color). **(B–D)** are graphs showing comparison of parameters analyzed in paired human myocardial samples from patients pre-LVAD placement (representing chronic heart failure) and post LVAD at the time of cardiac transplant (representing recovered myocardium). Each dot represents a patient. **(B)** Percentage of fibrosis is reduced in post LVAD samples in comparison to pre-LVAD myocardium; During the same time frame, **(C)** Number of endothelial cells have increased per low power field (100X, average of 5 fields) and **(D)** number of fibroblasts per low power field (100X, average of 5 fields) decreased. **(E)** Representative picture of the myocardial sample (100X) obtained at the time of LVAD implant showing interstitial fibrosis (blue) stained with trichrome. **(F)** Representative picture of the myocardial sample (100X) from the same patient shown in **(E)** obtained at the time of transplant representing recovery showing reduced fibrosis (blue trichrome staining).

With evidence of a role for cell fate transitions between endothelial and mesenchymal cells in heart failure and recovery we explored further the occurrence of such cell transitions in human heart failure. We studied 25 end stage heart failure myocardial samples for the presence of cells that showed expression of both endothelial (VE-Cadherin) and mesenchymal (FSP-1) markers. Mean age of the cohort was 55 years, 76% were males and 44% Caucasian and 48% had ischemic cardiomyopathy. Dual staining cells were evident in 72% of the samples (Figure 7A). To assess for cell transitions in the context of cardiac recovery in humans, we assessed the paired samples described above. As an indirect marker of cell transitions, the assessment of number of endothelial and fibroblast cells in these samples showed that the number of endothelial cells increased, and the number of fibroblasts decreased, from heart failure to recovery in parallel to a reduction of fibrosis (Figures 7B–F).

## Discussion

We present here evidence for a new conceptual framework for the mechanisms of recovery from heart failure. In a mouse model of heart failure recovery, and in human myocardial specimens from pre- and post-LVAD hearts, we find evidence for the role of cell fate transitions between endothelial and mesenchymal cells during the progression from normal to disease state of heart failure followed by a recovery of the disease state. This was associated with a reduction in fibrosis during morphological recovery from heart failure. To be sure, this new conceptual framework needs to be buttressed by additional work, but its recognition opens the door to a new vista of exploration and potentially, a novel therapeutic avenue.

The mechanisms of recovery from heart failure can be further elucidated in the mouse model we generated using pharmacologically-induced heart failure, where removal of the inducing agents is associated with echocardiographic recovery of cardiac function. This functional recovery is associated with evidence of histological improvement, with normalization of heart weight and myocyte size, together with a reduction in myocardial fibrosis. Furthermore, an analysis of global gene expression was consistent with reversal of heart failure, revealing a transcriptional profile in the recovered myocardium very similar to that of control mice and distinctly different from the heart failure transcriptional signature (Figure 2C).

A gene ontology analysis of the transcriptional data from the mouse model, through the continuum of heart failure and recovery suggested a role for endothelial and mesenchymal cell transitions (Figure 4). Of the pathways that were downregulated during heart failure and upregulated during recovery there were many that are relevant to endothelial and mesenchymal cell transitions. These pathways included “vasculogenesis involved in coronary vascular morphogenesis”; “positive regulation of epithelial cell migration”; “negative regulation of smooth muscle cell migration”; “epithelial tube formation” and “morphogenesis of embryonic epithelium.” EndoMT has been shown in lineage tracing studies in mice as an active contributor to cardiac fibrosis (Zeisberg et al., 2007) and suggested as a contributor of cardiac pathology (Kovacic et al., 2019) but the reverse phenomenon of MEndoT has only been postulated as an anti-fibrotic mechanism in the heart (Li et al., 2010) and its role remains controversial due to contradictory experimental findings (Ubil et al., 2014). The pathway analysis of the transcriptional data from the mouse model, together with histological evidence of reduced fibrosis, were consistent with a role for MEndoT in recovery from heart failure.

Many of the GO pathways listed in Figure 4B suggest EndoMT transitions with mechanism like cell motility, heart development, cell-cell junction assembly, positive regulation of SMAD protein import into nucleus, as well as transcriptional effectors of EndoMT such as NF-Kappa transcription factor activity and transforming growth factor beta receptor signaling pathway. TGF-B is well-recognized to promote EMT and EndoMT while some of the BMPs like BMP-7 and 4 are negative regulators of this process. The cellular mechanisms of both these signaling molecules are mediated by translocation of various SMADs into the nucleus (Valcourt et al., 2005). The top pathway that was upregulated in heart failure followed by a downregulation in recovery (Figure 4B) was the Wnt (wingless/integrated) signaling pathway. This pathway governs paracrine and intracellular communications through a group of signal transduction mechanisms categorized into canonical, non-canonical polarity and non-canonical Wnt/calcium pathways. This signaling pathway has an established role in carcinogenesis and embryogenesis where cell transitions have been more comprehensively described (Yao et al., 2011; Kim et al., 2014). It is possible that the upregulation of Wnt may support EndoMT and cardiac fibrosis during heart failure. Notably, some angiogenic processes appear to be upregulated during heart failure in our model. This may be a compensatory response to the pharmacological agents that are increasing cardiac work and myocardial hypertrophy. In chronic human heart failure, these compensatory processes may fail, explaining the increase in fibrosis and reduction in vascularity observed in pre-LVAD specimens.

In the murine model, many of the genes that are well established as promoters of EMT and EndoMT (e.g., SNAI1, SNAI2, and Twist) had increased expression in the HF phase, supporting a role for EndoMT in the disease state. Interestingly SNAI1 remained elevated during recovery. While SNAI1 has been associated as the genetic switch for EMT (Nieto, 2002) recent studies have postulated its role beyond EMT and as a promoter of cell plasticity and mobility in general (Barrallo-Gimeno and Nieto, 2005; Rowe et al., 2009; Wu and Zhou, 2010) and possibly contributes to promoting cell plasticity of various cell types during the recovery phase.

The GO pathway assessment of the gene changes from control to heart failure and heart failure to recovery showed that there are more pathways involved in the process of recovery (185 GO pathways up regulated and 384 down regulated) than progression to heart failure (95 GO pathways up regulated and 46 GO pathways down regulated) suggesting that cardiac recovery from non-ischemic heart failure is a complex, active process and not a passive reversal of the pathological changes that occurred during the injury phase. Also, while the global gene expression pattern was very similar in the recovered myocardium when compared to age matched controls, there are genes that were uniquely dysregulated during recovery suggesting that despite morphological normalization there are cellular mechanisms that might not revert to normalcy (supporting the notion of heart failure remission vs. complete recovery).

Another murine model of heart failure recovery was recently published using a transverse aortic constriction (TAC) approach, and permitting recovery from heart failure by debanding the aortic compression (Weinheimer et al., 2018). Of note, there is minimal fibrosis in the TAC model compared to extensive interstitial fibrosis in the angiotensin-II based models. Because interstitial fibrosis is an important pathological process in human heart failure, our model may provide new insights that are clinically relevant to the generation and reversal of fibrotic changes in human heart failure.

When the global transcriptional profiles of the mouse and human hearts were compared, we discovered a high correlation between murine and human genes that were differentially regulated during recovery. The similarity in the directionality in the gene set enrichment analyses comparing the changes in the mouse model to the pre and post-LVAD human samples along with the similarity in the specific EndoMT/MEndoT genes (Figure 5) suggests that the transcriptional changes mediating heart failure recovery may be similar in mouse and man. The relative change in the specific genes were not the same for some of the genes in the human samples when compared to the mouse. The differences we observed between mouse and man in the relative change in gene expression may be related to differences between species; between the chronicity and etiology of the heart failure; or the effects pf the pharmacological/device treatment of heart failure. Considering these confounding variables, it is remarkable that our mouse model replicates many of the functional, structural and transcriptional changes in heart failure and recovery in humans. Thus, it may be a predictive model for future investigations of recovery from heart failure. Previous investigators have studied the myocardium pre- and post-LVAD to elucidate the biology of recovery as best summarized by Yacoub and Terracciano (2011). In these patients with severe heart failure, chronic unloading of the heart with a LVAD reverses some of the adverse remodeling of the left ventricle, in association with a reduction in fibrosis (Drakos et al., 2011). In our analysis of paired myocardial samples pre- and post-LVAD, we observed a reduction in fibrosis together with an increase in endothelial cells and reduction of number of fibroblasts supporting MET. The histological evidence for dual-stained cells, demonstrating both endothelial and fibroblast markers in cells from the failing human myocardium, is intriguing and is consistent with cell transitions in human heart failure. This is the first description of such dual-staining cells in human heart failure. This observation raises the possibility of a partial endothelial to mesenchymal transition, a phenomenon suggested to play a role in angiogenesis (Welch-Reardon et al., 2015). Further studies are needed to delineate the origins and behaviors of specific contributions of various sub-types of cells. A recent single-cell reconstruction of the human heart revealed several subtypes of endothelial cells, and one particular subset that may play a specific role in heart failure and recovery (Wang et al., 2020). Future studies with single cell analysis of disaggregated hearts in a heart failure recovery model are needed to further characterize cellular subsets that contribute to reductions in fibrosis and/or vascular recovery.

There are several limitations to our study. First, the gene expression data is generated from the whole myocardium, which is a heterogenous population of cells including myocytes, fibroblasts, vascular smooth muscle, endothelial, and immune cells. Single cell analyses will define the alterations in the transcriptional profile of specific cell types during heart failure and will provide a refined understanding of which cell types are contributing to the observed transcriptional changes in the whole heart. Furthermore, lineage tracing studies will be critical in documenting a mesenchyme-to-endothelial transition. Such studies will require multiple transgenic lines as no one transgenic line will be definitive. Although our mouse model of recovery shares many of the functional, histological, and transcriptional features of human heart failure, it is a rather acute model, and there may be compensatory pathways that are active in this acute model that are not functioning in chronic human heart failure. While angiotensin-II increases blood pressure, our previous work indicates that the changes in the myocardium are not purely load related. Other studies have in fact demonstrated an adverse direct myocardial impact of angiotensin II independent of blood pressure (Regan et al., 2015).

## Conclusion

Cardiac recovery using current pharmacotherapies yields only a modest improvement of ejection fraction from 5 to 26% in a year (Goldfinger and Nair, 2014). While chronic unloading with a durable left ventricular assist device has shown promise of histological recovery by resting the heart, not more than 1% of LVAD patients are able to recover their hearts to a clinically meaningful degree in the real world. This is possibly due to lack of focused therapies promoting cardiac recovery. We have established a non-ischemic mouse model of heart failure recovery with demonstration of phenotypic and genotypic reversal from heart failure state and have correlated our pre-clinical findings with human heart failure specimens. Much more work needs to be done to confirm and expand upon our hypothesis, but the concept that EndoMT contributes to HF and MEndoT toward recovery from heart failure may lead to new pathophysiological insights and therapeutic avenues.

## Data Availability Statement

The original contributions generated for the study are publicly available. This data can be found here: https://www.ncbi.nlm.nih.gov/geo/query/acc.cgi?acc=GSE161309.

## Ethics Statement

The animal study was reviewed and approved by Houston Methodist Research Institute Ethics committee.

## Author Contributions

AB and KC conceived the project, designed the experiments, and interpreted the data. GW, AC, KY, and HM-A performed the data analysis and experiments. AB and GW wrote the manuscript with comments from AC, KY, HM-A, RT, JC, and KC. All authors contributed to the article and approved the submitted version.

## Conflict of Interest

The authors declare that the research was conducted in the absence of any commercial or financial relationships that could be construed as a potential conflict of interest.

## Supplementary Material

Supplementary Table 1 List of GO pathways representing genes that had expression up regulated in HF compared to control.

Supplementary Table 2 List of GO pathways representing genes that had expression down regulated in HF compared to control.

Supplementary Table 3 List of GO pathways representing genes that had expression down regulated in HF compared to recovery.

Supplementary Table 4 List of GO pathways representing genes that had expression was up regulated in HF compared to recovery.

## Abbreviations

HF: Heart Failure
EF: Ejection Fraction
EMT: Epithelial to Mesenchymal transition
EndoMT: Endothelial to mesenchymal transition
MEndoT: Mesenchymal to Endothelial transition
LVAD: Left Ventricular Assist device.
